# Implementation Burden and Hidden Labor in a Multisite Digital Psychiatry Trial

**DOI:** 10.3390/healthcare14111430

**Published:** 2026-05-22

**Authors:** Linda Rubene-Kesele

**Affiliations:** Department of Psychiatry and Narcology, Rīga Stradiņš University, LV-1007 Riga, Latvia; linda.rubene@rsu.lv

**Keywords:** implementation burden, digital solutions, psychiatry, mental health, multisite studies, hidden labor, healthcare systems

## Abstract

**Highlights:**

**What are the main findings?**

- Implementation burden is sustained by hidden, unrecognized labor at the site level.
- High performance at small research sites is achieved through intensified individual workload.

**What are the implications of the main findings?**

- Trial planning should explicitly account for implementation workload and staffing needs.
- Addressing hidden labor may improve sustainability and workforce well-being in digital trials.

**Abstract:**

Background: Multisite digital psychiatry trials increasingly rely on complex onboarding and implementation processes at local research sites. While outcome-focused evaluations are common, less attention has been paid to the site-level labor required to operationalize such studies in real-world settings, particularly at smaller or resource-constrained sites. This study addresses this gap by examining hidden implementation labor from a single-site reflexive perspective. Methods: This study adopts a reflexive qualitative case study approach to examine onboarding and implementation processes at a single research site participating in a multisite digital psychiatry trial (ClinicalTrials.gov: NCT04953208). The analysis draws on longitudinal experiential data, supported by site-specific documentation, onboarding timelines, troubleshooting records, device-management materials, data quality assurance activities, and internal communications generated during site coordination and implementation activities. Results: Five interrelated themes were identified: hidden labor and role overload; resource scarcity at small research sites; fragmented remote communication and technical coordination; multi-role professional contexts and competing demands; and the impact of external systemic disruptions. Findings show how administrative, technical, logistical, and coordination tasks were absorbed into individual roles, often exceeding initial role expectations. Despite limited resources, the site achieved high performance through intensified individual effort, masking the true implementation burden. This pattern is conceptualized as a high-performance paradox, in which apparent site efficiency may conceal substantial hidden labor and role compression. Conclusions: This site-level reflexive account highlights the central role of hidden labor in sustaining implementation in multisite digital psychiatry trials. Recognizing and explicitly resourcing implementation work, particularly at small research sites, may improve feasibility, sustainability, and equity across study settings. The study contributes a practice-based methodological perspective on how implementation burden can be identified through reflexive analysis of site-level trial processes.

## 1. Introduction

Digital mental health interventions are increasingly used to expand access to mental health care across diverse healthcare settings [1,2]. Within this broader shift, multisite digital psychiatry trials are important for evaluating how such interventions can be implemented across different local contexts [3,4]. While such trials are typically evaluated in terms of clinical outcomes and efficacy, the operational processes required to implement them at local research sites remain less well understood, particularly in resource-constrained environments.

This paper examines these operational processes from the perspective of a single research site participating in a multisite digital psychiatry trial. The focus is on implementation burden, understood here as the cumulative work required to translate a trial protocol into day-to-day site-level practice. Particular attention is given to hidden labor, role compression, and coordination work that may remain underrecognized in formal trial reports.

The aim of this study is to examine onboarding and implementation processes at a single research site using a reflexive qualitative case study approach, with a focus on identifying underrecognized sources of implementation burden. The study was guided by the following questions: What forms of hidden labor were involved in local onboarding and implementation? How did staffing constraints and role boundaries shape implementation burden? How did remote coordination and technical troubleshooting affect site-level work? What implications can be drawn for planning future multisite digital mental health trials?

The analysis draws on longitudinal experiential data generated in a multisite digital psychiatry trial (ClinicalTrials.gov: NCT04953208) [3,5] through site coordination, supported by site-specific documentation, onboarding timelines, and internal communications. While the study is grounded in a single-site context, it highlights mechanisms of hidden labor, role compression, and coordination complexity that may be relevant to other resource-constrained research settings. All examples are presented in anonymized form.

### 1.1. Literature Review

Multisite digital psychiatry and mental health intervention studies have expanded alongside advances in digital technologies and efforts to improve access to care across diverse settings [1,2,6,7,8]. Although such studies are often evaluated through clinical outcomes, recruitment, adherence, and efficacy, their success also depends on local implementation work. This includes onboarding, documentation, technical support, troubleshooting, and coordination across sites [9,10]. These site-level processes are often underreported, especially in publications focused primarily on clinical efficacy [11].

Implementation science literature has consistently identified several structural and organizational barriers in complex clinical studies, including staffing limitations, time pressure, competing institutional priorities, and coordination challenges across sites [6,10,11]. In digital mental health research, these barriers may be intensified by the need to manage technical infrastructure, platform usability, device logistics, and ongoing troubleshooting [6,9]. Qualitative studies examining trial staff perspectives have shown that recruitment, onboarding, and intervention delivery frequently require substantial informal labor that extends beyond formally defined roles [9,12].

This focus on implementation as practical and organizational work is consistent with Normalization Process Theory, which conceptualizes implementation as the work through which new practices are enacted, embedded, and integrated into healthcare settings. NPT has been used to study implementation processes across complex healthcare interventions and has been described as useful for examining how implementation is shaped by sense-making, participation, collective action, and reflexive monitoring [13,14]. However, the present study does not apply NPT as a formal coding framework; rather, it draws on this broader implementation science perspective to situate site-level implementation burden as a form of practical work.

Research on multisite and multinational clinical trials further emphasizes coordination burden as a recurring challenge [9,11,12,15]. Studies describing collaborative trials across institutions note that communication across geographic, institutional, and professional boundaries can be fragmented, leading to delays, misunderstandings, and increased reliance on site-level problem-solving [9,11,12,15]. Similarly, Morrow et al. described researcher experiences in a large multisite implementation trial, highlighting challenges related to bureaucracy, the distinction between implementation and clinical trials, COVID-19 disruption, balancing rigour and pragmatism, data access, and the need to streamline administrative and research procedures [16]. Such coordination demands are often managed through ad hoc solutions and individual effort, rather than through standardized or well-resourced support structures. From an implementation-as-work perspective, these demands can be understood as part of the collective action required to make a complex intervention operational in a specific setting [13]. Despite their importance, these forms of labor are rarely foregrounded in final trial reports [9].

A related body of literature addresses resource constraints and inequities between research sites. Smaller or less-resourced sites may face persistent human resource limitations, lack of redundancy in key roles, and reduced access to technical or administrative support [6,10,11]. However, apparent site efficiency does not necessarily indicate sufficient resources. Small sites may demonstrate high levels of recruitment or timely data delivery through intensified individual workload rather than structural advantage [9,11]. This may create a potential high-performance paradox: strong site-level performance may conceal considerable hidden labor and limited resilience. This interpretation also aligns with implementation theory emphasizing that successful implementation depends on the mobilization of social, cognitive, and structural resources across specific contexts, rather than on protocol design alone [14,17,18].

Methodologically, reflexive and practice-based qualitative approaches have been increasingly recognized as valuable for examining implementation processes in healthcare and research settings [19,20,21]. Reflexive thematic analysis emphasizes the active role of the researcher in interpreting patterns of meaning and developing themes, rather than treating themes as simply emerging from data [22]. Such approaches foreground contextual knowledge, experiential insight, and positionality, offering perspectives that complement formal process evaluations and quantitative metrics [6,9,10]. Reflexive accounts by clinicians, researchers, and coordinators provide important insight into the lived realities of implementing complex interventions, particularly in environments shaped by systemic constraints [9].

Despite these contributions, limited published work has explicitly examined how implementation burden is absorbed at the level of individual research sites within digital psychiatry trials, especially in smaller healthcare systems [6,9,10]. Previous studies have described implementation barriers, recruitment challenges, and coordination difficulties, but less attention has been paid to how these demands accumulate within local site roles and become managed through hidden labor and role compression. The present paper addresses this gap by offering a reflexive qualitative case study of onboarding and implementation processes at a Latvian study site. Its contribution lies in making visible the site-level labor that may sustain apparently successful implementation while remaining insufficiently represented in formal trial reporting.

### 1.2. Study Context and Setting

The present paper is situated within a multisite psychiatry study conducted under the DisCoVeR project. The trial investigated a combined digital intervention targeting cognitive and affective processes in individuals with major depressive disorder, incorporating computerized cognitive training and non-invasive brain stimulation, and was implemented across multiple research sites. The full study protocol, including detailed methodological descriptions, has been published previously (DisCoVeR protocol; ClinicalTrials.gov registration NCT04953208 with a registration date of 9 July 2021) and is therefore not reproduced here [3].

The Latvian site participated as one of the recruiting and implementing centers, with responsibilities defined in accordance with the published protocol. Recruitment and clinical monitoring of participants were conducted by site clinicians under the supervision of a local principal investigator. The reflections presented in this paper focus exclusively on the Latvian site and its onboarding and implementation processes.

Preparatory activities at the Latvian site began in 2019, including ethics submissions, translation and adaptation of study materials, and coordination of documentation, in which the author was actively involved. The author formally assumed the role of site coordinator in March 2020 and remained in this role through March 2024, and continues to be involved in data analysis and manuscript preparation related to the study. This timeframe is relevant because it covered several phases of local implementation, including study preparation, recruitment, participant onboarding, intervention delivery, troubleshooting, follow-up, and post-trial data quality assurance.

### 1.3. Author Role and Site Responsibilities

The author’s formal role at the Latvian site was that of site coordinator. In practice, this role encompassed a broad range of responsibilities related to local onboarding and implementation. These included organizing and maintaining site-specific documentation; supporting clinicians with study-related documentation; conducting participant onboarding sessions; providing technical support related to the digital intervention; and managing ongoing troubleshooting throughout the study. The role also involved coordinating communication between the Latvian site, other participating sites, and central study teams when clarification or problem-solving was required.

In addition, the author was responsible for coordinating the physical components of the study at the Latvian site, including the receipt, storage, distribution, and maintenance of study devices, as well as the secure handling and transfer of biological samples for centralized analysis. The author was also involved in local data quality assurance processes, including monitoring data completeness and participating in data cleaning activities. Parallel to these responsibilities, the author contributed to data analysis and manuscript preparation as part of doctoral research associated with the project.

Although the site coordination role was initially shared between two staff members, these responsibilities were assumed by the author alone during the later stages of the study. The Latvian site operated with a relatively small team but achieved rapid participant recruitment and data provision compared to other participating centers, some of which experienced recruitment delays. This combination of limited staffing, overlapping responsibilities, and accelerated implementation timelines constitutes an important contextual backdrop for the site-level reflections presented in this paper. It also illustrates why implementation work is examined here not only as protocol execution, but as the practical coordination, adaptation, and problem-solving required to make the trial operational at the local site.

### 1.4. Study Aim and Contribution

The aim of this study is to examine onboarding and implementation processes at a single research site participating in a multisite digital psychiatry trial, using a practice-based qualitative approach. By drawing on longitudinal involvement in site coordination, this analysis seeks to identify underrecognized sources of implementation burden, including hidden labor, role compression, and coordination challenges. The study is conceptually aligned with implementation perspectives that understand implementation as practical, relational, and organizational work required to embed complex interventions in healthcare settings [17,18]. It does not apply Normalization Process Theory as a formal coding framework, but uses this broader implementation-as-work perspective to situate the site-level labor required to operationalize a digital psychiatry trial. The study contributes a site-level perspective to the implementation literature, highlighting mechanisms that may influence feasibility, sustainability, and workforce demands in digital mental health research. These findings may inform the design and planning of future multisite digital psychiatry studies, particularly in resource-constrained healthcare settings.

## 2. Materials and Methods

This study adopts a reflexive qualitative case study approach to examine onboarding and implementation processes at a single study site participating in a multisite digital psychiatry trial. The analysis draws on longitudinal experiential data generated through the author’s involvement in site coordination and implementation activities throughout the study period. The unit of analysis was the implementation process at the Latvian study site, rather than participant-level clinical outcomes. No additional participants were recruited for the present reflexive analysis. This qualitative analysis is reported in accordance with established standards for reflexive qualitative research and process evaluations in clinical trials.

The analytic timeframe covered preparatory activities beginning in 2019, formal site coordination from March 2020 to March 2024, and continued involvement in post-trial data quality assurance, analysis, and manuscript preparation thereafter. This timeframe included several phases of local implementation: study preparation, recruitment, participant onboarding, intervention delivery, troubleshooting, follow-up, and post-trial data quality assurance.

Data sources included onboarding timelines, site-specific documentation, internal communications with clinicians, central study teams, and external partners, as well as records related to troubleshooting, device management, and data quality assurance. Documentation related to biological sample handling and device logistics was also included. These materials were not collected as a prospective qualitative dataset but were generated as part of routine trial coordination and implementation work. They were reviewed retrospectively and iteratively to support a structured reflexive analysis of implementation processes.

An inductive, thematic approach was used to organize reflections into core themes. First, the author repeatedly reviewed relevant site-level materials and reflexive notes developed during analysis to identify recurrent implementation challenges. Second, initial observations were grouped into provisional analytic categories related to workload, role boundaries, communication processes, resource constraints, and contextual disruptions. Third, these categories were compared across different phases of the study, including preparation, recruitment, onboarding, intervention delivery, troubleshooting, follow-up, and post-trial data quality assurance. Finally, candidate themes were refined to capture recurrent and implementation-relevant patterns related to implementation burden and hidden labor. Coding and theme development were conducted manually through repeated reading of site-level materials, annotation of recurring implementation challenges, grouping of related observations into provisional analytic categories, and iterative refinement of these categories into final themes.

This analytic approach was informed by reflexive thematic analysis, which treats theme development as an interpretive and iterative process. Themes were therefore understood not as objective categories extracted from the material, but as analytic constructions developed through repeated engagement with site-level materials, reflexive notes, and the study questions.

Themes were refined through repeated comparison of experiences across different phases of the study, with attention to how structural conditions, resource availability, and communication processes shaped implementation burden. Reflexivity was integral to the analytic process, with the author explicitly acknowledging the influence of her dual role as clinician-researcher and site coordinator on the observations presented. This dual position provided close access to implementation processes while also introducing the possibility of subjective interpretation. To address this, the analysis was supported by repeated engagement with site documentation, reflexive memoing, comparison across implementation phases, and explicit attention to the boundaries of what could and could not be inferred from a single-site perspective.

This approach aligns with established reflexive methodologies in implementation science and health services research, where experiential and contextual knowledge is recognized as a valuable source of insight into real-world research processes. The study did not include triangulation through interviews with additional staff, participants, or other study sites. The findings should therefore be interpreted as a contextualized reflexive account rather than as a universally generalizable evaluation of multisite digital psychiatry trials.

## 3. Results

### 3.1. Hidden Labor and Role Overload

A central theme emerging from the site-level reflection was the extent of hidden labor embedded in onboarding and implementation processes. While the formal role at the Latvian site was defined as site coordination, the practical demands of implementation extended far beyond this designation. Responsibilities accumulated across administrative, technical, logistical, and coordination domains, often without clear delineation or redistribution of tasks. Many of these activities, such as repeated troubleshooting, informal problem-solving, and ad hoc coordination between stakeholders, were essential for study continuity but remained largely invisible within formal project structures.

This pattern was identified through repeated evidence across site documentation, internal communications, troubleshooting-related records, and reflexive notes. Examples included clarifying documentation procedures for clinicians, supporting participant onboarding, coordinating technical problem-solving, following up on unresolved device issues, and maintaining communication between local and central study teams. These tasks were not isolated events but recurring components of implementation work.

This role expansion intensified over time, particularly during later stages of the study when staffing at the site was reduced. As responsibilities became increasingly concentrated, the distinction between defined roles and emergent needs blurred, resulting in sustained role overload. The reliance on individual flexibility and informal labor functioned as a key enabling factor for implementation but also represented a significant, underacknowledged burden.

### 3.2. Resource Scarcity at Small Research Sites

Implementation processes at the Latvian site were shaped by persistent resource constraints, particularly in terms of human resources. The site operated with a small core team, limiting opportunities for task sharing, redundancy, or coverage during periods of increased demand. This theme was supported by site-level materials and reflections indicating limited staff redundancy, concentration of coordination tasks, and continued recruitment and data-management responsibilities despite restricted personnel capacity. In this context, even routine implementation tasks required careful prioritization and negotiation of limited capacity.

Despite these constraints, the site achieved rapid participant recruitment and timely data provision compared to other participating centers. However, this performance was sustained through intensive individual effort rather than structural support. Resource scarcity thus did not manifest as inefficiency but rather as increased personal workload and reduced buffering against disruptions. This dynamic highlights how high-performing small sites may carry disproportionate implementation burden that remains obscured in aggregate project reporting.

This pattern is interpreted here as a high-performance paradox: operational success at a small site may conceal the intensified individual labor required to produce that success. In this case, rapid recruitment and data provision did not indicate the absence of implementation burden. Instead, they reflected the concentration of implementation work within a limited number of individuals and the absence of substantial staffing redundancy.

### 3.3. Fragmented Remote Communication and Technical Coordination

Another prominent theme concerned the challenges of fragmented, predominantly remote communication across institutional and organizational boundaries. Coordination with external partners, particularly device manufacturers, relied on asynchronous communication, which complicated troubleshooting, device repair, and replacement processes. The absence of direct, in-person contact limited opportunities for rapid resolution and mutual understanding of technical issues.

These communication barriers had downstream consequences for both participants and site staff. Participants were sometimes required to make repeated visits to the study site to address technical problems, disrupting intervention continuity and increasing participant burden. At the same time, site coordinators assumed intermediary roles, translating technical information between parties and managing delays. This added layer of coordination further contributed to implementation workload and reinforced the centrality of informal labor in sustaining the study.

Across implementation materials, technical issues appeared not only as isolated device problems but as coordination events requiring communication between participants, local clinicians, central study teams, and external partners. The labor involved was therefore both technical and relational: it required identifying the problem, communicating it across organizational boundaries, translating responses into actionable steps, and maintaining participant engagement while resolution was pending.

### 3.4. Multi-Role Professional Contexts and Competing Demands

Implementation at the Latvian site took place within a broader professional context characterized by multi-role employment and competing institutional demands. None of the personnel involved in the study worked exclusively on the project. Clinicians and research staff simultaneously balanced clinical duties, teaching responsibilities, research activities, and work across public and private healthcare settings. This theme was informed by site-level reflections and documentation showing that study tasks were carried out alongside clinical, academic, research, and institutional responsibilities outside the project. This pattern reflects common structural features of the Latvian medical system, where role multiplicity is normative rather than exceptional.

As a result, study-related tasks were frequently embedded within already full schedules, limiting the availability of protected time for onboarding and implementation activities. Implementation work therefore competed with other professional obligations, increasing cognitive and logistical load. This context amplified the impact of unforeseen challenges and reduced flexibility in responding to implementation disruptions.

This theme helps explain why apparently manageable implementation tasks could become burdensome in practice. Tasks such as responding to participant questions, resolving documentation issues, or arranging technical support were rarely difficult in isolation. Their burden arose from accumulation, timing, and competition with other clinical, academic, and institutional responsibilities.

### 3.5. External Systemic Disruptions During Implementation

Finally, implementation unfolded against a backdrop of broader external crises that introduced additional strain and uncertainty. This theme was identified through reflections and communications related to pandemic-related workflow adaptations and consortium-level disruption during the recruitment period. The COVID-19 pandemic emerged during active recruitment and onboarding phases, necessitating rapid adaptations to workflows, safety procedures, and participant interactions. These changes increased complexity and unpredictability in implementation processes.

In addition, geopolitical instability affecting another study site during the recruitment phase had indirect effects on the wider project team. Although the Latvian site was not directly exposed to these events, awareness of colleagues operating under conditions of war contributed to emotional and organizational strain within the consortium. These experiences underscore how multisite studies remain vulnerable to global disruptions that extend beyond local contexts and can influence implementation dynamics even at geographically distant sites.

In the present analysis, external crises were interpreted as amplifiers of existing implementation burden rather than as separate contextual events. They intensified coordination demands, increased uncertainty, and reduced the predictability of study workflows. This finding suggests that multisite digital trials require not only technical and procedural planning, but also contingency planning for system-level disruptions that may affect local implementation capacity.

## 4. Discussion

Rather than mapping findings onto a predefined implementation framework, this analysis adopts an interpretive approach to examine how implementation burden is produced and managed within a specific organizational context. The interpretation is conceptually aligned with implementation perspectives that understand implementation as practical, relational, and organizational work. The following discussion situates these findings in relation to existing implementation literature.

### 4.1. Key Contribution and Relation to Existing Literature

The most salient implementation challenge observed at the Latvian site was the extent of multitasking and the breadth of skills required to sustain onboarding and implementation, which substantially exceeded initial role expectations. This finding is consistent with prior literature suggesting that digital and multisite trials impose significant operational and coordination burdens on site staff, including time pressure, role strain, and reliance on informal problem-solving. However, the present reflexive account adds nuance by illustrating how these burdens accumulate within single individuals when staffing is limited, leading to pronounced role compression across administrative, technical, clinical, logistical, and analytical domains.

The specific contribution of this manuscript is therefore not simply that implementation barriers exist, but that the burden of resolving these barriers may become concentrated within local site roles. This is particularly important for small research sites, where limited staffing and reduced role redundancy can make implementation success dependent on informal, individualized labor. While previous studies acknowledge implementation burden in general terms, the concentration of heterogeneous responsibilities within one or two individuals and its implications for sustainability and well-being remains underemphasized. This site-level perspective highlights that successful implementation may depend less on formal role design than on unanticipated skill acquisition and continuous multitasking by individual actors.

### 4.2. Hidden Labor and Role Compression in Digital Psychiatry Trials

Hidden labor appears particularly salient in digital psychiatry trials due to the hybrid nature of the work involved. Such studies require not only clinical and research competencies but also technical literacy, coordination with external technology partners, and real-time troubleshooting within participant-facing contexts. The findings suggest that role compression, where multiple specialized tasks are absorbed into a single role, can distort assumptions about feasibility and cost by masking the true human resources required for implementation. While project timelines and budgets may appear adequate on paper, they often fail to account for the informal labor needed to bridge gaps between protocol design and real-world execution. This observation extends existing implementation literature by demonstrating how digital complexity may amplify reliance on individual adaptability rather than institutional support, thereby shifting risk and workload onto site personnel.

### 4.3. Resource Scarcity and the High-Performance Paradox at Small Sites

The experience of the Latvian site underscores a paradox that may be overlooked in multisite research: small or resource-constrained sites may demonstrate high performance, such as rapid recruitment and timely data delivery, precisely because of intensified individual effort rather than structural advantage. This pattern can be understood as a high-performance paradox: apparent site efficiency may conceal substantial hidden labor, role compression, and reduced resilience to disruption. While prior literature acknowledges disparities in resources across sites, this account highlights that such performance may be maintained through sustained overextension rather than scalable processes, raising concerns about long-term feasibility and equity between sites.

This interpretation has implications for how feasibility and site capacity are assessed in multisite trials. Recruitment speed or timely data delivery should not be treated as sufficient indicators of implementation feasibility unless accompanied by assessment of staffing resilience, role distribution, and workload sustainability.

### 4.4. Communication Technologies and the Human Interface

The reliance on remote and asynchronous communication across sites and external partners emerged as a critical factor shaping implementation burden. Although digital trials presume technological efficiency, this study illustrates how communication breakdowns, particularly with device manufacturers or technical support teams, can propagate delays, increase participant burden, and require substantial intermediary work by coordinators.

These findings align with existing concerns about coordination complexity in multisite trials but add specificity regarding the human labor required to translate between technical, clinical, and organizational domains. The results suggest that digital infrastructure does not eliminate coordination work but redistributes it, often invisibly. Future digital trials should therefore define technical escalation pathways before recruitment begins, including clear responsibilities for device troubleshooting, response timelines, and communication channels between sites, central teams, and external technology providers.

### 4.5. Systemic Context, External Shocks, and Implementation Fragility

Implementation processes were further shaped by broader systemic and geopolitical disruptions, including the COVID-19 pandemic and conflict affecting other study sites. While the impact of such events on research productivity has been widely acknowledged, this account highlights how external shocks may compound existing implementation burden by increasing uncertainty, emotional load, and coordination demands. Even when not directly experienced at a given site, awareness of crises elsewhere in a multisite consortium can influence morale, communication, and workflow, underscoring the interconnected vulnerability of large collaborative studies.

These findings suggest that contingency planning should be treated as part of implementation planning rather than as an exceptional response to crisis. For small sites, contingency planning should include backup staffing arrangements, flexible timelines, and procedures for maintaining participant support during disruptions.

### 4.6. Strengths, Limitations, and Implications for Future Research and Practice

A key strength of this study lies in its reflexive, site-level perspective, which provides detailed insight into implementation processes that are rarely captured in outcome-focused publications. By foregrounding experiential and contextual knowledge, the paper complements existing implementation research and highlights areas requiring greater structural attention.

Limitations include its focus on a single site and reliance on reflective analysis rather than formal qualitative interviews, which constrains generalizability. The analysis reflects the perspective of one author who was directly involved in site implementation. Although the analysis was supported by site-level documentation, onboarding timelines, communications, troubleshooting records, and data quality assurance materials, it was not triangulated through interviews with additional staff, participants, or other study sites. Subjectivity and recall bias therefore cannot be excluded. The findings should be interpreted as a contextualized reflexive account rather than as a generalizable evaluation of multisite digital psychiatry trials.

Nonetheless, the findings suggest important implications for future digital psychiatry research, including the need for more explicit resourcing of implementation roles, recognition of hidden labor, clearer delineation of responsibilities, and contingency planning for systemic disruptions. More specifically, future multisite digital mental health trials should consider allocating protected coordinator time, budgeting for technical troubleshooting and documentation work, defining backup staffing arrangements, establishing clear escalation routes for technical problems, and including site-level implementation workload in feasibility assessments. Addressing these factors may improve sustainability, equity, and transparency in multisite digital mental health trials.

## 5. Conclusions

This site-level reflexive account highlights how onboarding and implementation in a multisite digital psychiatry trial may depend on substantial, often unrecognized labor at the local level. The findings suggest that implementation burden is not solely a function of protocol complexity or technological demands but may also be shaped by role compression, resource scarcity, and the accumulation of diverse responsibilities within individual actors. While such labor can enable high performance and timely delivery, it frequently remains invisible within formal project structures, potentially distorting assumptions about feasibility, cost, and sustainability.

By foregrounding the lived realities of implementation at a small research site, this paper contributes contextualized insight to the digital mental health and implementation science literature. Recognizing and explicitly resourcing the human work required to bridge protocol design and real-world execution may improve equity between sites, protect staff well-being, and enhance the long-term viability of complex digital interventions. Because this analysis is based on a single-site reflexive design, the findings should not be interpreted as universally generalizable. Instead, they offer practice-based insights that may be transferable to similar resource-constrained research settings. Greater attention to site-level experiences, including vulnerability to external disruptions, may support digital psychiatry research that is both effective and sustainable.

## Data Availability

No new datasets were generated or analyzed in this study. The analysis is based on reflexive, practice-based qualitative insights derived from the author’s involvement in study implementation; therefore, data sharing is not applicable.

## Abbreviations

The following abbreviations are used in this manuscript:

MDD	Major depressive disorder
NPT	Normalization Process Theory

## References

[1] Lattie E.G., Stiles-Shields C., Graham A.K. (2022). An overview of and recommendations for more accessible digital mental health services. Nat. Rev. Psychol..

[2] Philippe T.J., Sikder N., Jackson A., Koblanski M.E., Liow E., Pilarinos A., Vasarhelyi K. (2022). Digital Health Interventions for Delivery of Mental Health Care: Systematic and Comprehensive Meta-Review. JMIR Ment. Health.

[3] Dechantsreiter E., Padberg F., Morash A., Kumpf U., Nguyen A., Menestrina Z., Windel F., Burkhardt G., Goerigk S., Morishita T. (2023). Examining the synergistic effects of a cognitive control video game and a home-based, self-administered non-invasive brain stimulation on alleviating depression: The DiSCoVeR trial protocol. Eur. Arch. Psychiatry Clin. Neurosci..

[4] Pyle M., Loftus L., Emsley R., Freeman D., Gillard S., Gumley A., Sierpatowska J., Wood L., O’Connor R.C., Pfeiffer P. (2024). Study protocol for an adaptive, multi-arm, multi-stage (MAMS) randomised controlled trial of brief remotely delivered psychosocial interventions for people with serious mental health problems who have experienced a recent suicidal crisis: Remote Approaches to Psychosocial Intervention Delivery (RAPID). Trials.

[5] Dechantsreiter E., Padberg F., Rubene L., Benjamini Y., Goerigk S., Hummel F., Lotan A., Bavelier D., Rancans E., Nahum M. (2025). Combining tDCS with a cognitive control video game training in depression: A multicenter randomized controlled trial (DiSCoVeR). Brain Stimul..

[6] Berardi C., Antonini M., Jordan Z., Wechtler H., Paolucci F., Hinwood M. (2024). Barriers and facilitators to the implementation of digital technologies in mental health systems: A qualitative systematic review to inform a policy framework. BMC Health Serv. Res..

[7] Krukowski R.A., Ross K.M., Western M.J., Cooper R., Busse H., Forbes C., Kuntsche E., Allmeta A., Silva A.M., John-Akinola Y.O. (2024). Digital health interventions for all? Examining inclusivity across all stages of the digital health intervention research process. Trials.

[8] Jenkins C.L., Imran S., Mahmood A., Bradbury K., Murray E., Stevenson F., Hamilton F.L. (2022). Digital Health Intervention Design and Deployment for Engaging Demographic Groups Likely to Be Affected by the Digital Divide: Protocol for a Systematic Scoping Review. JMIR Res. Protoc..

[9] Allan S., Mcleod H., Bradstreet S., Bell I., Whitehill H., Wilson-Kay A., Clark A., Matrunola C., Morton E., Farhall J. (2021). Perspectives of Trial Staff on the Barriers to Recruitment in a Digital Intervention for Psychosis and How to Work Around Them: Qualitative Study Within a Trial. JMIR Hum. Factors.

[10] Hafner J., Tokgöz P., Dockweiler C. (2025). Implementation Factors of Digital Health Interventions in Depression Care—The Perspective of Health Professionals. Healthcare.

[11] Forjuoh S.N., Helduser J.W., Bolin J.N., Ory M.G. (2015). Challenges Associated with Multi-institutional Multi-site Clinical Trial Collaborations: Lessons from a Diabetes Self-Management Interventions Study in Primary Care. J. Clin. Trials.

[12] Sappor E.E., Chakraborty R. (2025). Exploring Staff Perceptions of the Management of Clinical Trials and Its Impact on Enhancing Health Service Delivery. Hospitals.

[13] McEvoy R., Ballini L., Maltoni S., O’Donnell C.A., Mair F.S., MacFarlane A. (2014). A qualitative systematic review of studies using the normalization process theory to research implementation processes. Implement. Sci..

[14] May C.R., Cummings A., Girling M., Bracher M., Mair F.S., May C.M., Murray E., Myall M., Rapley T., Finch T. (2018). Using Normalization Process Theory in feasibility studies and process evaluations of complex healthcare interventions: A systematic review. Implement. Sci..

[15] Peralta G., Sánchez-Santiago B. (2024). Navigating the challenges of clinical trial professionals in the healthcare sector. Front. Med..

[16] Morrow A., Tyedmers E., Debono D., Chan P., Steinberg J., Tiernan G., Hogden E., Taylor N. (2025). Trials and tribulations: A qualitative exploration of researcher perspectives on navigating the challenges of health system implementation research. BMJ Open.

[17] Nilsen P., Bernhardsson S. (2019). Context matters in implementation science: A scoping review of determinant frameworks that describe contextual determinants for implementation outcomes. BMC Health Serv. Res..

[18] May C.R., Johnson M., Finch T. (2016). Implementation, context and complexity. Implement. Sci..

[19] Rana K., Poudel P., Chimoriya R. (2023). Qualitative Methodology in Translational Health Research: Current Practices and Future Directions. Healthcare.

[20] Renjith V., Yesodharan R., Noronha J.A., Ladd E., George A. (2021). Qualitative Methods in Health Care Research. Int. J. Prev. Med..

[21] Hamilton A.B., Finley E.P. (2019). Qualitative Methods in Implementation Research: An Introduction. Psychiatry Res..

[22] Byrne D. (2022). A worked example of Braun and Clarke’s approach to reflexive thematic analysis. Qual. Quant..

